# A development of assistant surgical robot system based on surgical-operation-by-wire and hands-on-throttle-and-stick

**DOI:** 10.1186/s12938-016-0189-7

**Published:** 2016-05-20

**Authors:** Myungjoon Kim, Chiwon Lee, Woo Jung Park, Yun Suhk Suh, Han Kwang Yang, H. Jin Kim, Sungwan Kim

**Affiliations:** Interdisciplinary Program for Bioengineering, Graduate School, Seoul National University, Seoul, 110-744 South Korea; Institute of Medical and Biological Engineering, Seoul National University, Seoul, 151-742 South Korea; Department of Surgery, Seoul National University College of Medicine, Seoul, 110-799 South Korea; Department of Mechanical & Aerospace Engineering, Seoul National University College of Engineering, Seoul, 151-742 South Korea; Department of Biomedical Engineering, Seoul National University College of Medicine, Seoul, 110-799 South Korea

**Keywords:** Assistant surgical robot system, Minimally invasive surgery (MIS), End-effector of surgical robot, Novel master interface (NMI), da Vinci research kit (dVRK), Surgical-operation-by-wire (SOBW), Hands-on-throttle-and-stick (HOTAS)

## Abstract

**Background:**

Robot-assisted laparoscopic surgery offers several advantages compared with open surgery and conventional minimally invasive surgery. However, one issue that needs to be resolved is a collision between the robot arm and the assistant instrument. This is mostly caused by miscommunication between the surgeon and the assistant. To resolve this limitation, an assistant surgical robot system that can be simultaneously manipulated via a wireless controller is proposed to allow the surgeon to control the assistant instrument.

**Methods:**

The system comprises two novel master interfaces (NMIs), a surgical instrument with a gripper actuated by a micromotor, and 6-axis robot arm. Two NMIs are attached to master tool manipulators of da Vinci research kit (dVRK) to control the proposed system simultaneously with patient side manipulators of dVRK. The developments of the surgical instrument and NMI are based on surgical-operation-by-wire concept and hands-on-throttle-and-stick concept from the earlier research, respectively. Tests for checking the accuracy, latency, and power consumption of the NMI are performed. The gripping force, reaction time, and durability are assessed to validate the surgical instrument. The workspace is calculated for estimating the clinical applicability. A simple peg task using the fundamentals of laparoscopic surgery board and an in vitro test are executed with three novice volunteers.

**Results:**

The NMI was operated for 185 min and reflected the surgeon’s decision successfully with a mean latency of 132 ms. The gripping force of the surgical instrument was comparable to that of conventional systems and was consistent even after 1000 times of gripping motion. The reaction time was 0.4 s. The workspace was calculated to be 8397.4 cm^3^. Recruited volunteers were able to execute the simple peg task within the cut-off time and successfully performed the in vitro test without any collision.

**Conclusions:**

Various experiments were conducted and it is verified that the proposed assistant surgical robot system enables collision-free and simultaneous operation of the dVRK’s robot arm and the proposed assistant robot arm. The workspace is appropriate for the performance of various kinds of surgeries. Therefore, the proposed system is expected to provide higher safety and effectiveness for the current surgical robot system.

## Background

Conventional minimally invasive surgery (MIS) has become one of the most advocated surgical operation approach because it offers benefits such as low blood loss, reduced time to drain removal, shorter hospital stay, better pain score, fewer follow-ups, smaller incision, and reduced complication rate than open surgery [1, 2]. However, MIS has the following disadvantages: (i) operating time is relatively longer than that of conventional open surgery and (ii) because the degrees of freedom (DOFs) of the surgical instrument is low, surgical operations such as suturing are difficult for inexpert surgeons to perform, resulting in the need for highly-trained surgeons to perform surgical operations [3, 4]. Consequently, robot-assisted laparoscopic surgery has been developed to overcome the limitations of both types of surgeries [5–8]. Introduction of the surgical robot has resulted in benefits such as shorter operating time, reduced blood loss, less surgeon fatigue, better pain score, reduced time to drain removal, shorter hospital stay, reduced complication rate, and fewer follow-ups, even compared with conventional MIS [1]. Furthermore, it facilitates improved surgical precision, better visualization, and more intuitive and ergonomic instrument control—resulting in shorter learning curves for surgeons [9].

The da Vinci surgical robot (Intuitive Surgical, Inc., Sunnyvale, CA, USA), one of the most advanced surgical robots, has been used in 1.5 million laparoscopic surgical operations globally over the past decade [10]. Nevertheless, there remain some issues to be resolved. One such issue is the collision that sometimes occurs between the operation robot arms and the assistant instrument during robotic surgery, which can cause injury to patients [9, 11–13]. This collision can be caused by an inexperienced assistant or miscommunication and misaligned intent between the surgeon and the assistant [14, 15]. Several solutions have been proposed. For example, a manipulator with a relatively small mass, which reduces the collision force, and force-feedback system has been proposed [12]. A surgery simulator for real-time collision processing and visualization that is able to prevent several types of collisions has also been developed [16]. A novel surgical robot design that minimizes the operating envelope during surgery has also been proposed [17]. In the proposed design, the operating envelope is minimized to help the assistant to work alongside the robot, and also results in fewer collisions during surgery. A fourth arm that the operating surgeon can utilize for key steps and maneuvering during operations has also been proposed for the da Vinci surgical robot system [18]. This system avoids collisions between the operating robot arm and the assistant’s instrument by turning over control of the assistant’s instrument to the surgeon. Although these systems have been proposed partially based on the issue of collision between the operating robot arm and the assistant’s instrument, they have several deficiencies: (i) they are limited to simulation and cannot be directly applied to the surgical robot system [16], (ii) they can only minimize or reduce, not prevent, collisions [12, 17], and (iii) the surgeon cannot simultaneously manipulate both the assistant robot arm and the operation robot arm, resulting in discontinuous surgical operation [18]. In addition, this assistant robot arm cannot perform surgical operations such as removal of resected tissue because it cannot move outside the incision.

This paper proposes an assistant surgical robot system that overcomes these issues. The system, which consists of an assistant robot arm and its wireless controller, aims to remove the cause of collisions due to tiredness or miscommunication and misaligned intent between the surgeon and the assistant by allowing the surgeon to control the assistant instrument. Further, a wireless controller is developed for simultaneous control of the operating robot arm and the assistant robot arm, thereby preventing discontinuous surgical operation. The assistant robot arm consists of 6-DOFs external robot arm and a surgical instrument developed based on the surgical-operation-by-wire (SOBW) concept that has been reported in our previous study [2, 19]. SOBW was inspired by the fly-by-wire (FBW) system in aerospace engineering, in which the wing control is based on electrical wires for reliable control [20], instead of a mechanical wires [21–24]. The concept is applied in the medical field with the mechanical strings in the surgical robot system replaced with electrical wires. In this sense, all the motions of the proposed assistant robot arm, including the external robot arm and the surgical instrument, are actuated by electrical actuators such as alternating current servo motors and micromotor. Further, the yawing and pitching motions are removed from the surgical instrument as they are not necessary for the performance of dexterous movements. In exchange, the diameter of the proposed surgical instrument is 6 mm. This is smaller than that of the most extensively used da Vinci surgical robot system’s 8 mm EndoWrist. The rolling, translational, and fulcrum point motions of the surgical instrument are performed by the 6-DOFs external robot arm. The gripping motion is achieved by converting the rotational motion of the micromotor into translational motion using male and female screws, with the female screw linked to the gripper. Consequently, the gripping force can be controlled by adjusting the position of the micromotor. The durability of the surgical instrument developed was verified via a 1000 times of repeated durability tests. A da Vinci research kit (dVRK), donated by Intuitive Surgical, Inc., was used in this study to perform as the operation robot arm system. The dVRK is a research kit consisting of several parts, including master tool manipulators (MTMs), patient side manipulators (PSMs), stereo viewer, and foot pedal, from the first generation da Vinci surgical robot system. A novel master interface (NMI), a wireless communication interface for the assistant robot arm, was developed to simultaneously control the assistant robot arm and the operation robot arm in order to avoid the surgeon having to stop the operation robot arm to manipulate the assistant robot arm. The NMI is based on the hands-on-throttle-and-stick (HOTAS) controller, which is widely used in aerospace for flight control [2]. The concept of HOTAS controller has been reported in our previous study [2, 25]. In this study, a multi-way switch and a wireless microprocessor were used to reflect the surgeon’s decision. Further, the NMI developed is relatively small and can easily be attached to the MTMs of the dVRK system for easy access when the surgeon is manipulating the MTMs. The accuracy, latency, and power consumption of the developed NMI were verified by repeated experiments. Simple peg tasks using the assistant robot arm system were also performed to evaluate the clinical applicability of the proposed assistant robot arm system. In addition, an in vitro test of semi-automatic resected object removal was conducted using the proposed assistant surgical robot system and the dVRK system to examine the performance of the proposed system. The results indicate that this novel surgical robot system can be effectively utilized for laparoscopic robotic surgery.

## Methods

The surgical robot system developed to overcome the limitations stated above comprises four parts: (i) dVRK system to perform as the operation robot, (ii) surgical instrument with the diameter of 6 mm, (iii) 6-axis external robot arm that provides translational, fulcrum point, and rolling motions, and (iv) two NMIs that respectively reflect the surgeon’s decision to control the external robot arm and the surgical instrument.

**Fig. 1:**
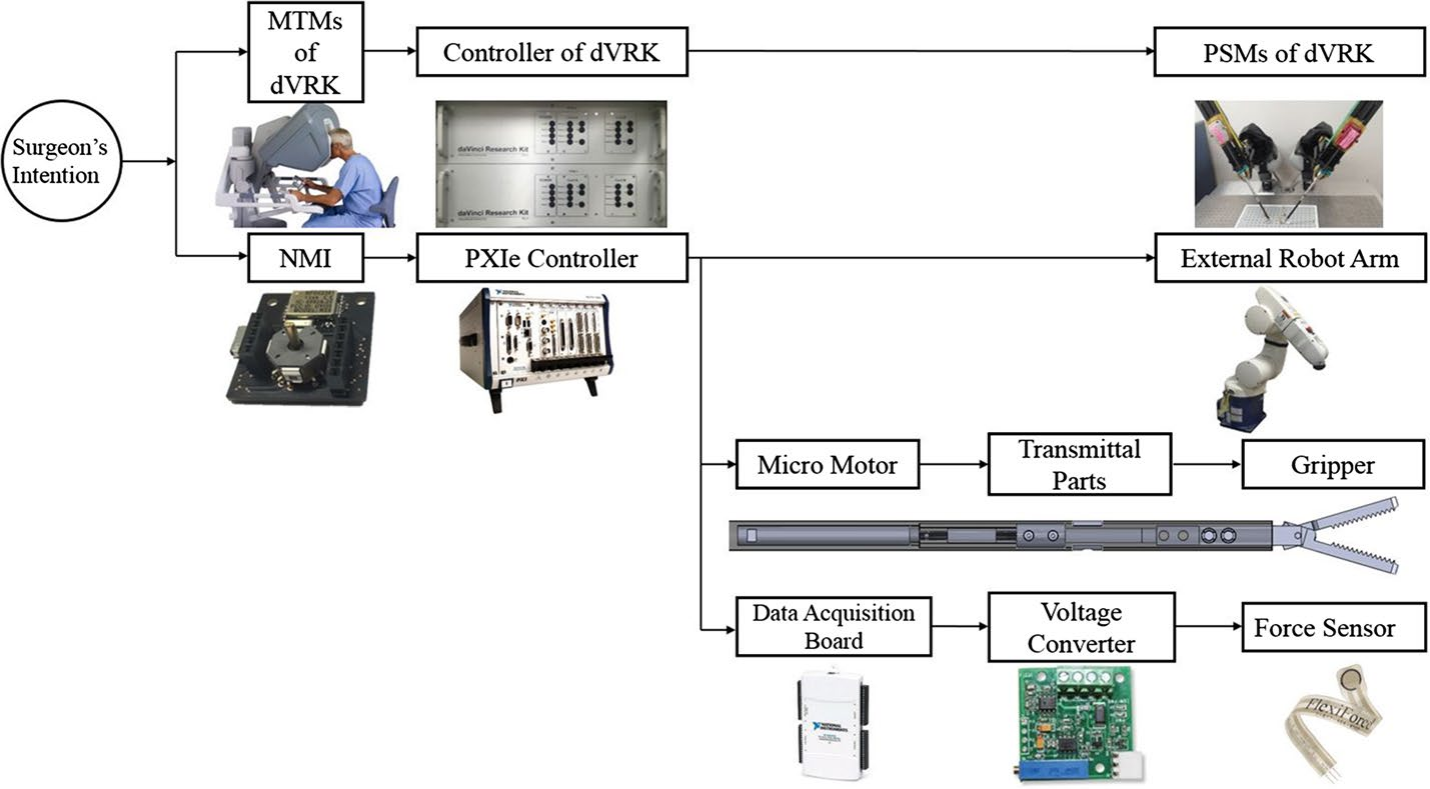
Control flow of the proposed assistant robot system driven by the surgeon’s intention. Software integration is based on the LabVIEW^Ⓡ^ software

These parts, with the exception of the dVRK system, were integrated via the LabVIEW^Ⓡ^ and the PXIe controller (LabVIEW^Ⓡ^ 2013, PXIe-8135 and 1062Q, National Instruments, Austin, TX, USA, Used valid license). The control flow of the overall system is illustrated in Fig. 1. As shown in the figure, the surgeon can simultaneously control both PSMs—the operation robot arms and the assistant robot arm developed—by manipulating the two MTMs and the two NMIs. Continuous surgical operation can thus be ensured via this control flow. The gripping motion of the surgical instrument is facilitated using an electronically commutated micromotor with the diameter of 4 mm (EC-4 motor, EPOS2 controller, Maxon Motor, Brünigstrasse, Sachseln, Switzerland), which is able to rotate in both clockwise and counterclockwise direction.

### da Vinci research Kit

**Fig. 2:**
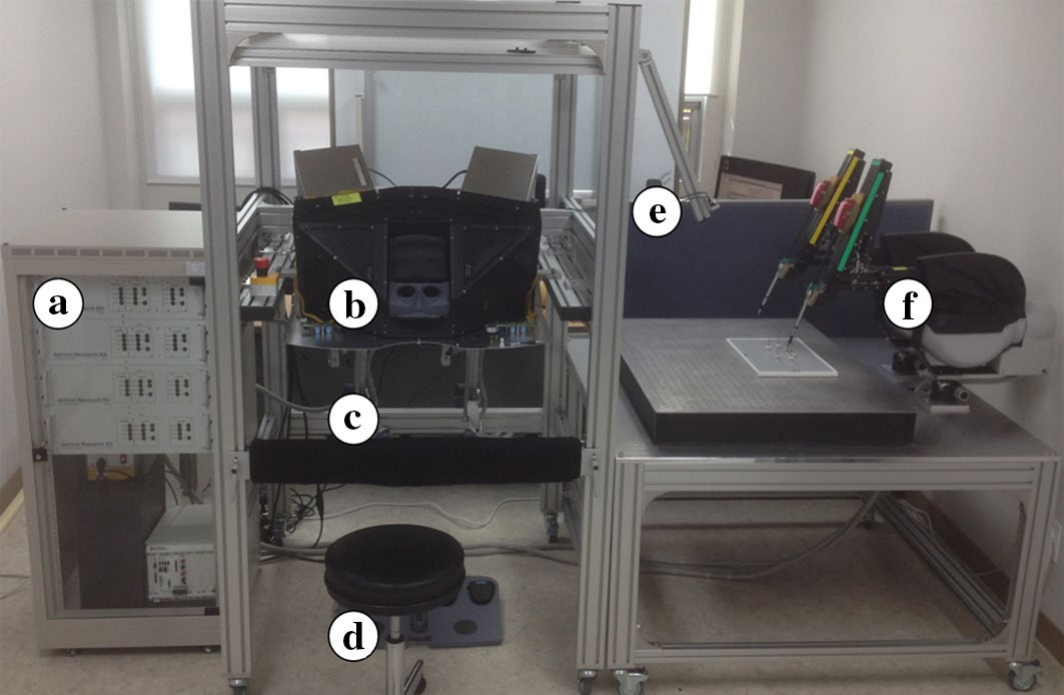
Overall system of the da Vinci research kit (dVRK). **a** Controllers. **b** Stereo viewer. **c** Master tool manipulators (MTMs). **d** Foot pedal. **e** Two webcams for providing images. **f** Patient side manipulators (PSMs). dVRK is used as operation surgical robot system in this research

The dVRK system was used to perform as an operation surgical robot system. The dVRK system comprises one foot pedal, two MTMs, two PSMs, and one stereo viewer to provide a three-dimensional stereo view for the user, as shown in Fig. 2. Two webcams are installed to provide images. Each MTM is able to manipulate its respective PSM during laparoscopic surgery. The dVRK system was integrated with the assistant surgical robot system.

### Surgical instrument

**Fig. 3:**
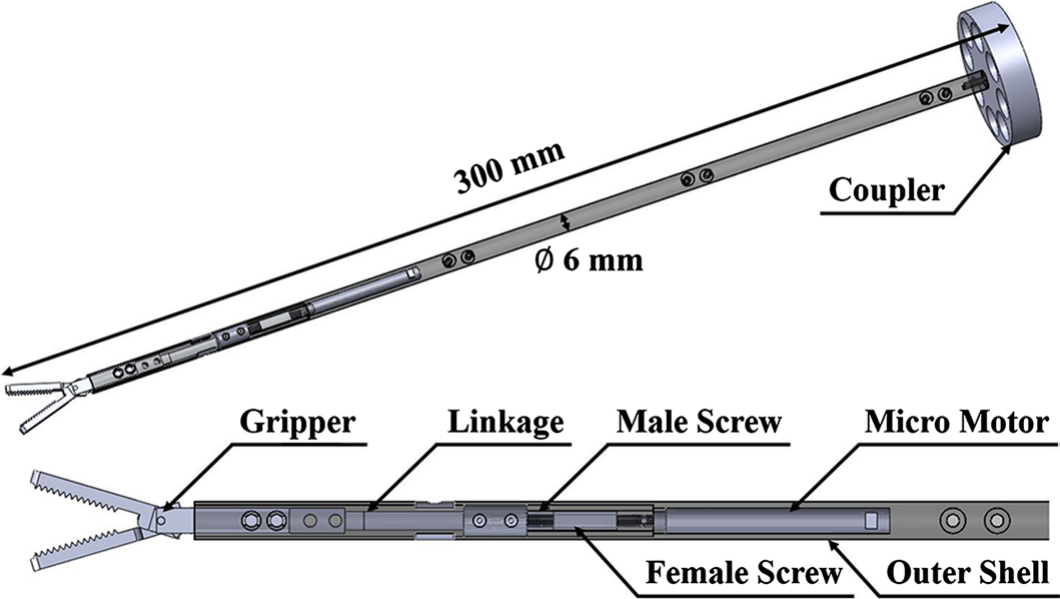
Design of the proposed surgical instrument. The gripping motion is achieved by converting the micro motor’s rotation motion into linear motion by male and female screw and linking the gripper with the female screw through the linkage. The length and the diameter of the surgical instrument is designed as 300 mm and 6 mm, respectively

**Fig. 4:**
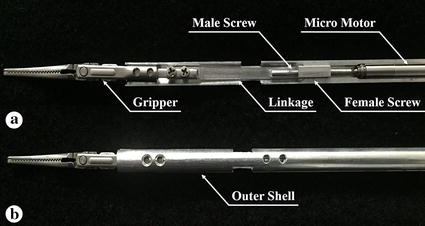
Surgical instrument manufactured using aluminum. **a** The surgical instrument without the *upper outer shell*. Actual position of the micro motor, male and female screw, linkage, and the gripper is shown. **b** The surgical instrument with the *upper outer shell*

A surgical instrument, which can perform only gripping motion, was developed specifically for the laparoscopic surgical robot system. Yawing and pitching motions were removed as they are not necessary for the assistant surgical instrument to perform dexterous motion. In exchange, the diameter of the proposed surgical instrument is 6 mm. This is smaller than that of the most extensively used da Vinci surgical robot system’s 8 mm EndoWrist and comparable with that of the 6 mm EndoWrist which has less applications. The rolling motion of the surgical instrument is achieved by the external robot arm by installing the instrument as an end-effector. Unlike other systems [7, 26], the surgical instrument can be easily replaced during surgery. The actuating force of the surgical instrument’s gripping motion is generated by converting the rotation of the micromotor’s shaft into translational motion using a male and female screw arrangement similar to the ball screw mechanism. Actuation of the micromotor causes the male screw to rotate around a fixed axis and the female screw to consequently move translationally along a straight line guided by the outer shell. Linear motion of the linkage is enabled by linking the female screw with the linkage. Further, the gripping motion is then generated by linking the linkage and the gripper, which was cut off from a laparoscopic forceps (Laparoscopic forceps, Ethicon Endo-Surgery, Cincinnati, Ohio, USA) and modified by adding a hole for the connection. This was possible because each side of the gripper is based on the slider crank mechanism, which can convert linear motion to rotational motion as a reciprocating pump engine. Each side of the gripper is aligned symmetrically with respect to the longitudinal axis, and thus can be actuated by the linear motion of the linkage simultaneously. Therefore, open and close motion of the gripper are then achieved by clockwise and counterclockwise rotation of the micromotor’s shaft. The overall design and the actual image of the surgical instrument are illustrated in Figs. 3 and 4. The outer shells and the other parts of the surgical instruments, such as the male and female screws, and the linkage were manufactured using aluminum and assembled with the micromotor and the gripper, as shown in Fig. 4. The surgical instrument was designed to be 300 mm long for surgical usability.

### External robot arm

**Fig. 5:**
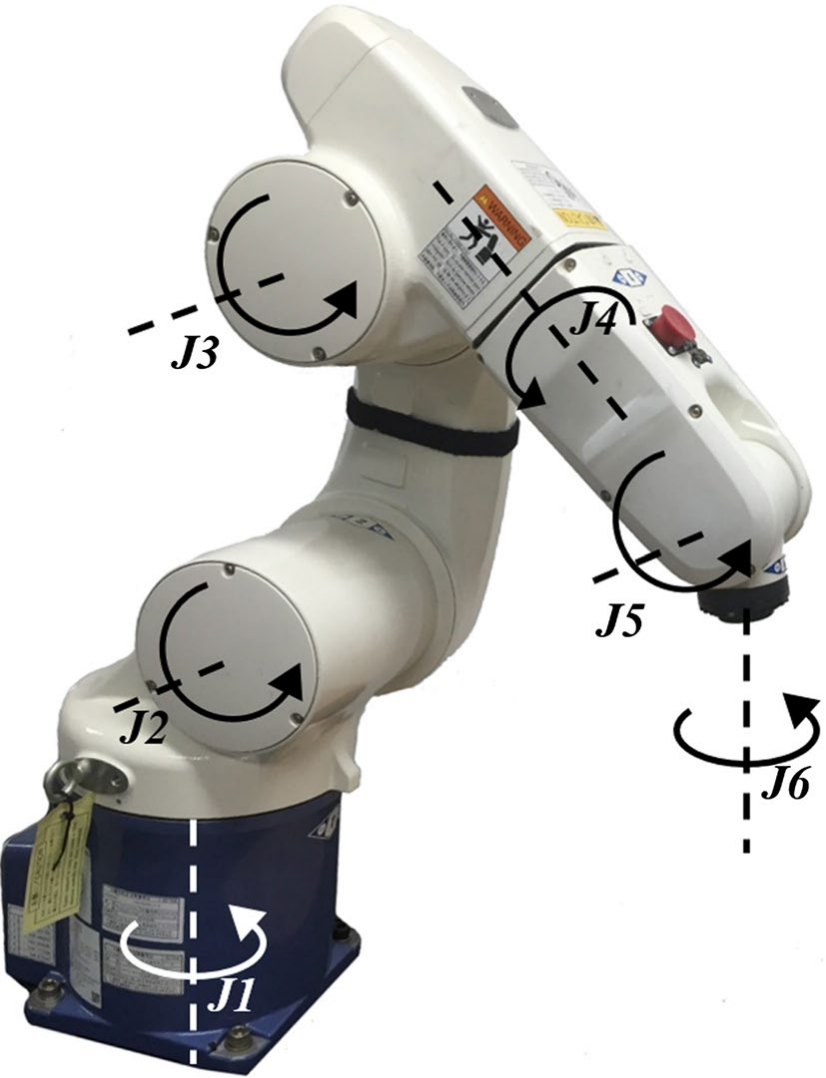
Joint information of the 6-degrees of freedom (DOFs) external robot arm. The fulcrum point motion and the translational motion of the surgical instrument are achieved by complex combination from *J1* to *J5*. The surgical instrument’s rolling motion is achieved by *J6*

An external robot arm (VS-6556G, DENSO, Kariya, Aichi Prefecture, Japan) with six joints, from *J1* to *J6*, is used to perform the surgical instrument’s translational, fulcrum point, and rolling motions, as shown in Fig. 5. The translational and the fulcrum point motions are achieved by coordinating joints *J1* to *J5* and controlling them based on the tool coordinates system, which sets the origin of the external robot arm to the origin of the end-effector. To perform the fulcrum point motion, a virtual remote center of motion (RCM) algorithm was applied to the external robot arm since it did not employ a RCM mechanism as the PSM of the dVRK system did. Thus, the RCM point of the external robot arm can be adjusted by the virtual RCM algorithm. As for the rolling motion, unlike the da Vinci surgical robot system’s Endowrist which can perform rolling motion by itself [27], it is achieved by joint *J6* of the external robot arm. The forward kinematics of the external robot arm has been described in previous work [2].

### Novel master interface (NMI)

**Fig. 6:**
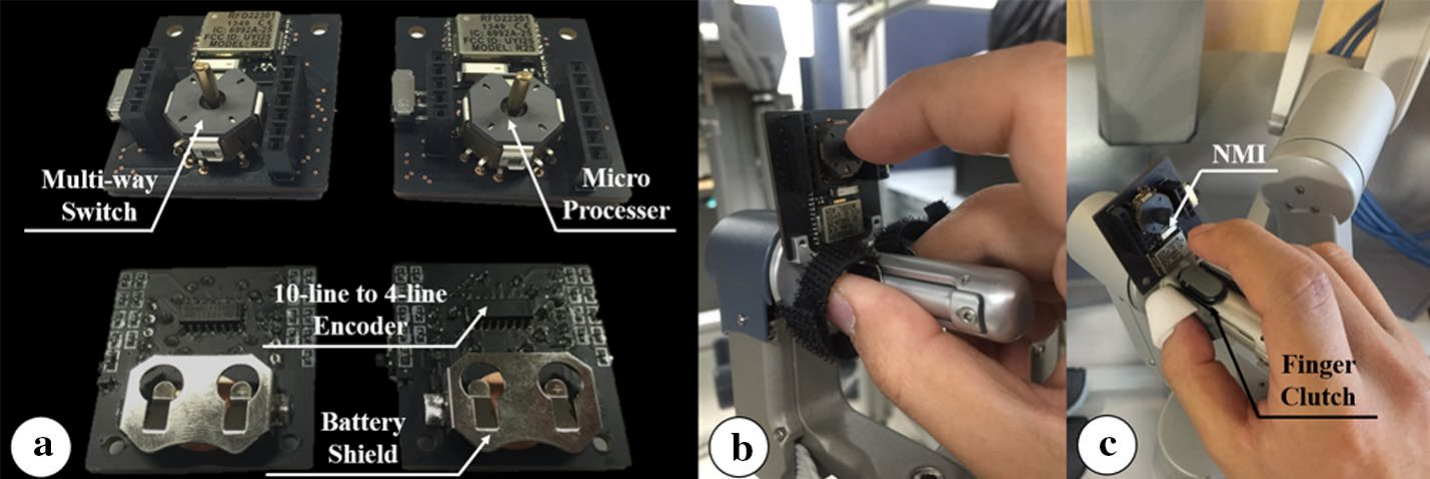
Developed novel master interface (NMI). **a** *Front* and *back* side of the NMI. **b** The NMI attached on the MTM of the dVRK system using the special holder. **c** Usage of the index finger to operate finger clutch and the NMI

**Fig. 7:**
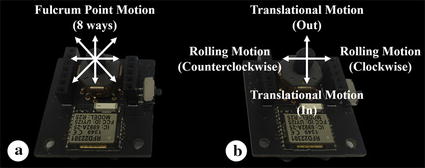
Mapping information between the NMI and the surgical instrument. **a** *Left* NMI. **b** *Right* NMI

The NMI—a wireless communication interface—was developed to carry out the surgeon’s intent as regards control of the assistant robot arm. The NMI was designed based on HOTAS, using a multi-way switch (RKJXL100401 V, ALPS, Tokyo, Japan)—more specifically, it has eight ways with a center push—for the surgeon to manipulate. In addition to the multi-way switch, the NMI comprises one Li-MnO_2_ type Lithium button cell battery (CR2032, Panasonic, Osaka, Japan), one 10- to 4-line encoder (CD40147B, Texas Instruments, Dallas, TX, USA), one Arduino-based microprocessor with a Bluetooth low energy radio frequency module (RFD 22301, RFduino, Hermosa Beach, CA, USA), and several resistors and capacitors to constitute the circuit. The output signal for each way of the multi-way switch is encoded as a four-digit number, representing a possible decision by the surgeon, via the 10- to 4-line encoder. On entering the Bluetooth module the four-digit number is sent to the wireless data receiver, where it is recognized as a command by the controller that manipulates the surgical instrument and the external robot arm based on the received signal. A circuit for this purpose was designed and implemented on a printed circuit board, and then assembled with other parts, as shown in Fig. 6a. To manipulate the NMIs simultaneously with MTMs, the two NMIs are tightly attached to two MTMs of the dVRK system using a special holder as shown in Fig. 6b. The reason for this is to not interrupt the operation of finger clutch of MTM which exists from the da Vinci Si system [28], and allow the surgeon to control the NMI using the index finger which is not used for manipulating the MTM, except for operating the finger clutch, as shown in Fig. 6c. Each NMI has dimensions 33 × 35 mm to ensure that they do not disrupt the motion of the MTMs. Figure 7 shows the mapping information between the NMI and the surgical instrument. NMI attached to the left MTM manipulates the fulcrum point motion, whereas that attached to the right MTM operates the translational and rolling motions. The gripping motion can be controlled by the center push of both left and right NMI. Thus, the translational, fulcrum point, and rolling motions, in addition to the gripping motion of the assistant robot arm can be simultaneously controlled with PSMs by manipulating the two NMIs and MTMs.

## Results

### Novel master interface

#### Accuracy test

The accuracy of the NMI was evaluated by intercepting the data it transferred using a specific LabVIEW^Ⓡ^ algorithm. The data transferred in both directions, along with the center push of the NMI were measured for 50 separate trials. No error occurred during these trials, indicating that the NMI can reflect the surgeon’s decision with high precision.

#### Data transfer time

The data transfer time of the NMI was determined by physically connecting it to the universal serial bus port to enable it to send data via wired communication. Then, the NMI transferred data both to the wireless data receiver and the universal serial bus port. The respective reception time for the data transferred through the two media types was each recorded using LabVIEW^Ⓡ^. This experiment was repeated 10 times. The resulting data transfer time for both media types was found to be 132 ms on average with a standard deviation (SD) of 5 ms.

#### Power consumption

Because the NMI is to be used during surgery, the amount of power it consumes has to be considered. As outlined above, the NMI utilizes a Li-MnO_2_ type Lithium button cell battery. To estimate the power consumption of the NMI, a LabVIEW^Ⓡ^ algorithm that continuously received data from the NMI and which recorded the time when the NMI stops the data transfer—inferring that the NMI was out of power—was developed. This experiment was executed 10 times. The results indicate that the NMI operated for 185 min (SD: 9 min), which is longer than the average time for several types of robotic surgeries [1, 4, 29, 30]. Moreover, because the button cell battery of the NMI can be easily replaced with a new one, for surgeries that extend beyond the time duration of the NMI, this would cause minimum inconvenience. Furthermore, the system would be safe even when the NMI has run out of battery since it would not send any data that can control the assistant robot system.

### Surgical instrument

#### Gripping force

A flexible piezoresistive sensor (Flexiforce, Tekscan Inc., South Boston, MA, USA), which is widely used in medical applications, was used to measure the gripping force of the proposed surgical instrument. The Flexiforce has been demonstrated to possess linearity [31]. Six precision weights (50, 100, 200, 500, 1000, and 2000 g) were used to calibrate the Flexiforce and transform the unit of the Flexiforce’s output signal from voltage to Newton. These weights were measured using the Flexiforce in order 10 times based on LabVIEW^Ⓡ^, and the output data were converted to force using the MATLAB linear regression method (MathWorks, Natick, MA, USA, using Seoul National University Academic License). Equation (1) shows the result of linear regression between the output voltage values and the force values:

1 $$F_{f} = (1592.70 \times {\text{V}} - 52.00)\, \times \,9.81$$

where V (V) is the output voltage of the Flexiforce and F_f_ (N) is the converted force value. To calibrate the measurement data and remove artifacts caused by the environment, the initial 500 sets of data were collected and processed in each experiment. The measurement data for the gripping forces were recorded using a data acquisition board (USB-6212 DAQ, National Instruments, Austin, TX, USA). Following data acquisition, a Savitzky-Golay filter was applied to filter out sharp noise in the measured signal using MATLAB [32].

**Fig. 8:**
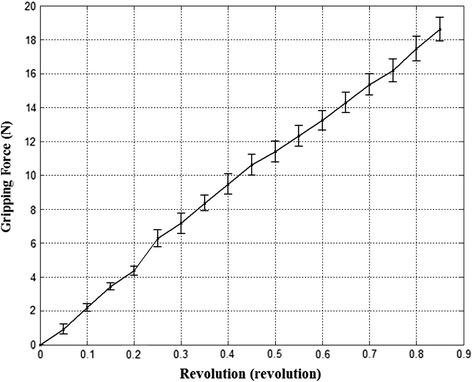
Experimental results of gripping force compliant with position of the micro motor. The experiments repeated 10 times and the standard deviation is plotted as *error bar*. The interval of the position of the micro motor was 0.05 revs

The gripping force was measured for every 0.05 revolution of the micromotor and the process repeated 10 times. Table 1 and Fig. 8 shows the results of the relationship between gripping force of the surgical instrument and the revolution of the micromotor. The mean of all gripping forces’ SD was computed as 0.51 N. The measurement data exhibited good linearity as the equation below:

2 $$F_{G} = {\text{c}}_{1} \times {\text{R}} + {\text{i}}_{1}$$

where F_G_ (N) is the gripping force and R (rev) is the revolution of the micromotor. The coefficients: c_1_ and i_1_ of Eq. (2) were identified as 21.70 and 0.31, respectively. The coefficient of determination was calculated as 1.00. In this experiment, it was assumed that the physical properties of the Flexiforce and tissue are similar and thus the force applied on them would be also comparable.

**Table 1:** Repeated experimental results of gripping force measurements

Revolution of the micro motor (rev)	Gripping force (N)	
	Mean	Standard deviation
0	0	0
0.05	0.92	0.30
0.10	2.00	0.11
0.15	3.63	0.21
0.20	4.37	0.16
0.25	6.27	0.70
0.30	7.27	0.60
0.35	8.36	0.46
0.40	9.47	0.60
0.45	10.61	0.62
0.50	11.41	0.62
0.55	11.93	0.31
0.60	13.24	0.57
0.65	14.30	0.60
0.70	15.35	0.62
0.75	16.18	0.66
0.80	17.46	0.73

#### Reaction time

The reaction time of the surgical instrument’s gripping motion was estimated by performing a step function using its gripping force value. The performance result was then compared with the ideal step function after applying the Savitzky-Golay filter for the same reason as described above. For this experiment, a gripping force value of 4.37 N (SD: 0.16 N) was selected because performing the highest gripping force value for the purpose of the experiment is meaningless. The experiment was repeated 10 times with a 2 s time interval between every two gripping motions and the time duration of the gripping motion. For the experiment, a specific LabVIEW^Ⓡ^ algorithm was developed to ensure that the intervals between the gripping motions were precise. The results obtained show that the step function generated by the gripping motion and the ideal step function have close conformability. The calculated mean of the time delay was 0.4 s.

#### Durability test

To test the durability of the surgical instrument, a LabVIEW^Ⓡ^ algorithm that continuously repeated the gripping motion was developed. The time intervals between every two gripping motions and the time duration of each gripping motion were set to 1 s. A gripping force of 4.37 N (SD: 0.16 N) was also selected in this experiment for the same reason as in the reaction time experiment. The gripping motion was repeated 1000 times and the gripping force values during the repetitions recorded. The mean gripping force was found to be 4.23 N with SD of 0.13 N, which is within the SD of the initial gripping force value.

### Workspace

**Fig. 9:**
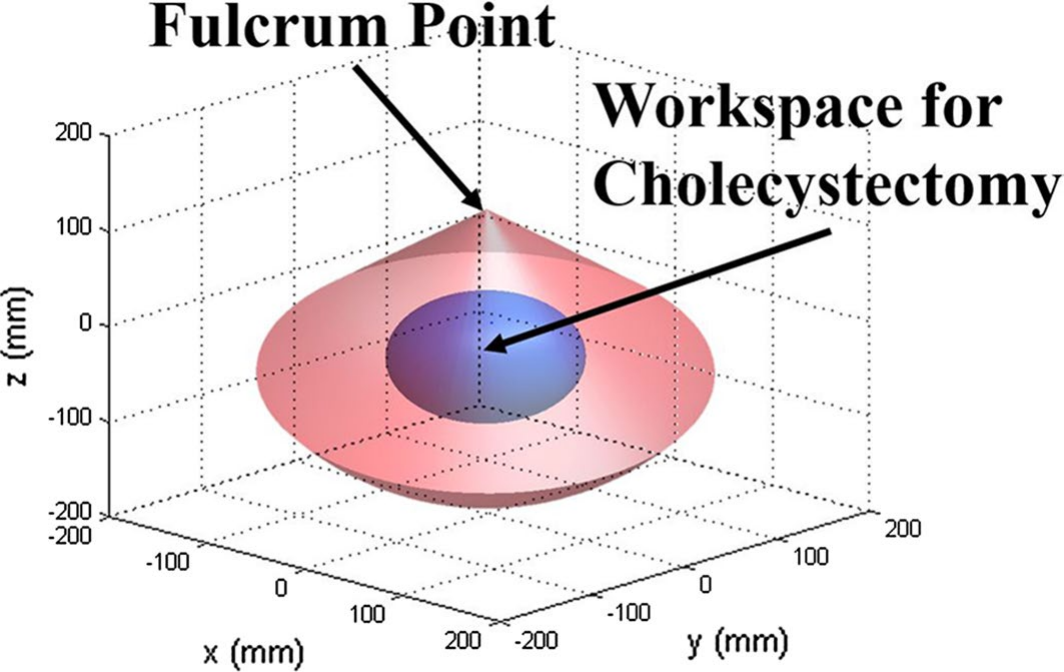
Workspace of the proposed assistant robot arm system

The workspace of the proposed additional surgical robot arm system was calculated using the external robot arm’s Denavit-Hartenberg parameter, inferred in previous research [2], and compared with the workspace required for cholecystectomy, as shown in Fig. 9. The calculated workspace was 8397.4 cm^3^, which exceeds the reference workspace [33], 549.5 cm^3^.

### Simple peg task

**Fig. 10:**
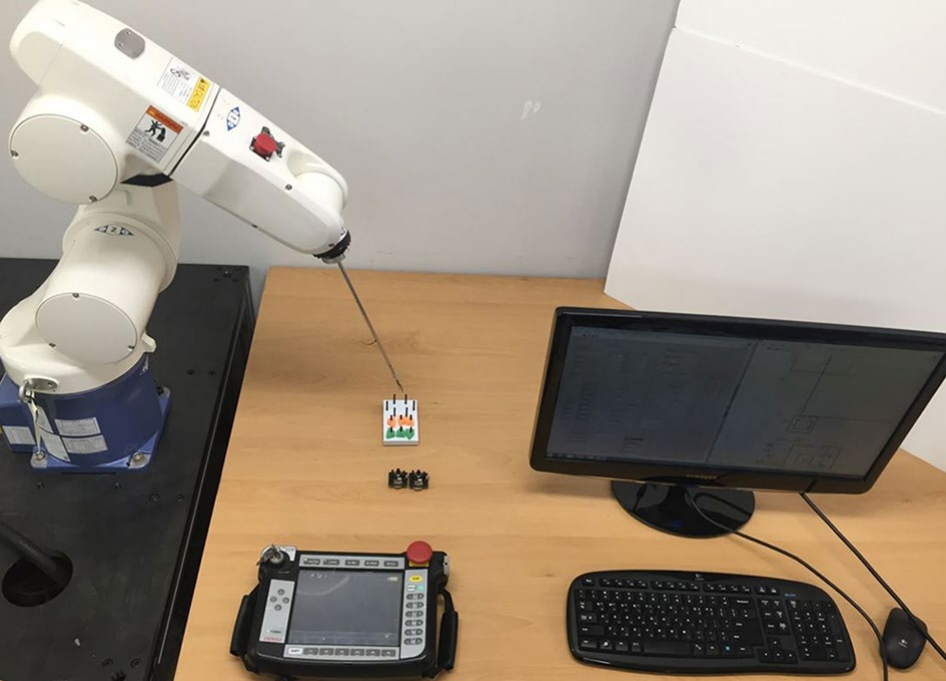
System setup for the peg transfer task using fundamental of laparoscopic surgery (FLS)

To validate the peg transfer performance, fundamentals of laparoscopic surgery (FLS) peg transfer kit were used in the performance of a modified block transfer task which followed standard FLS curriculum except for the mid-air transfer since only one surgical instrument was used in this research. The system setup is shown in Fig. 10. For this experiment, three novice volunteers were recruited. They were asked to follow the process outlined below for the modified FLS block transfer task curriculum—already predefined in previous research for validation of surgical robot systems and measurement of the surgeon’s technical skills and eye-hand coordination during surgery [2, 34–36]. The process can be divided into the following two steps: (i) the volunteers were asked to transfer six objects from the left side of the board to the right side of the board and (ii) the time taken to transfer the six objects, between the volunteer picking up the first object and releasing the last object, was measured. The time limit was set to 300 s which was determined by FLS curriculum, the same as in other research [2, 34–36]. The three volunteers executed three tasks each and the results show that the mean time of the peg transfer task was 250 s with SD of 6 s, as summarized in Table 2. According to Table 2, all volunteers succeeded in the peg transfer task within the time limit, 300 s.

**Table 2:** Execution time of block transfer task

Trial number	Volunteer 1	Volunteer 2	Volunteer 3	Total mean
1	242	268	260	257
2	231	261	253	248
3	226	264	246	245
Mean	233	264	253	250
SD	8	4	7	6

*SD* standard deviation

### In vitro test of semi-automatic resected object removal

**Fig. 11:**
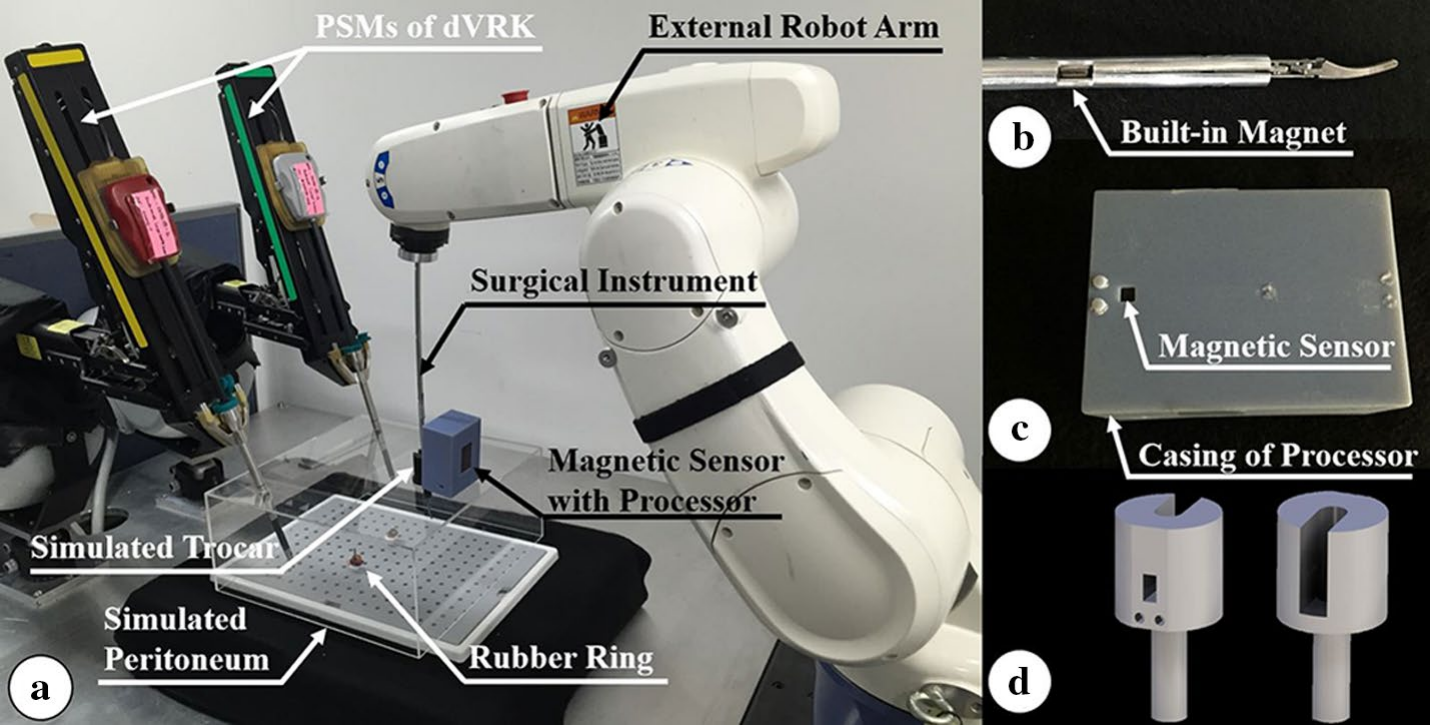
Setup for the in vitro test of semi-automatic resected object removal. **a** Overall system setup. **b** Built-in magnet of the surgical instrument to generate magnetic field. **c** Magnetic sensor with its controller board within the special housing. **d** Developed simulated trocar used in the in vitro test

To evaluate the performance of the assistant robot system as an assistant, which is the main purpose of this study, an in vitro test of semi-automatic resected object removal was performed. The setup for the test is depicted in Fig. 11. Three volunteers were recruited to perform the in vitro test. The process was as follows: (i) grasp and cut the rubber ring via the operation robot arm, (ii) then, grasp the cutted rubber ring using the assistant robot arm in order to remove the resected object from inside the simulated peritoneum and (iii) once the surgical robot had grasped the object, the assistant robot arm would switch to automatic mode to automatically take the object out of the simulated peritoneum. After the end of the surgical instrument was outside of the simulated peritoneum, it should put down the object and return to the operation area, and enable the volunteer to maneuver the assistant robot arm.

To achieve the automatic mode described in the third step of the test, a built-in magnet was installed within the outer shell of the surgical instrument and located 6 cm away from the tool tip of the surgical instrument, as shown in Fig. 11b, and a magnetic sensor (WSH138-XPAN2, Winson, Taiwan) that could linearly transform the detected magnetic force into voltage value was attached to the simulated trocar, as shown in Fig. 11c. An Arduino-based microcontroller board (ATmega328, Atmel, San Jose, CA, USA) was used to receive the voltage data transformed by the magnetic sensor, and a special housing was manufactured to attach the microcontroller board to the simulated trocar, as shown in Fig. 11c. The simulated trocar was developed to install the magnetic sensor and enable the surgical instrument to get rid of the resected object, as shown in Fig. 11d. To convert the data from voltage to distance, the voltage value was measured using the magnetic sensor for every 0.2 cm of the distance between the magnet and the magnetic sensor and the process repeated 10 times. As a result, the measurement data showed good linearity as the equation below:

3 $${\text{D}} = {\text{c}}_{2} \times {\text{V}} + {\text{i}}_{2}$$

where D (cm) is the distance between the magnet and the magnetic sensor and V (mV) is the voltage value measured by the magnetic sensor. The coefficients: c_2_ and i_2_ of Eq. (3) were identified as −0.10 and 70.10, respectively. The coefficient of the determination was calculated as 1.00. The maximum distance that can be sensed by the magnetic sensor is 2 cm.

**Fig. 12:**
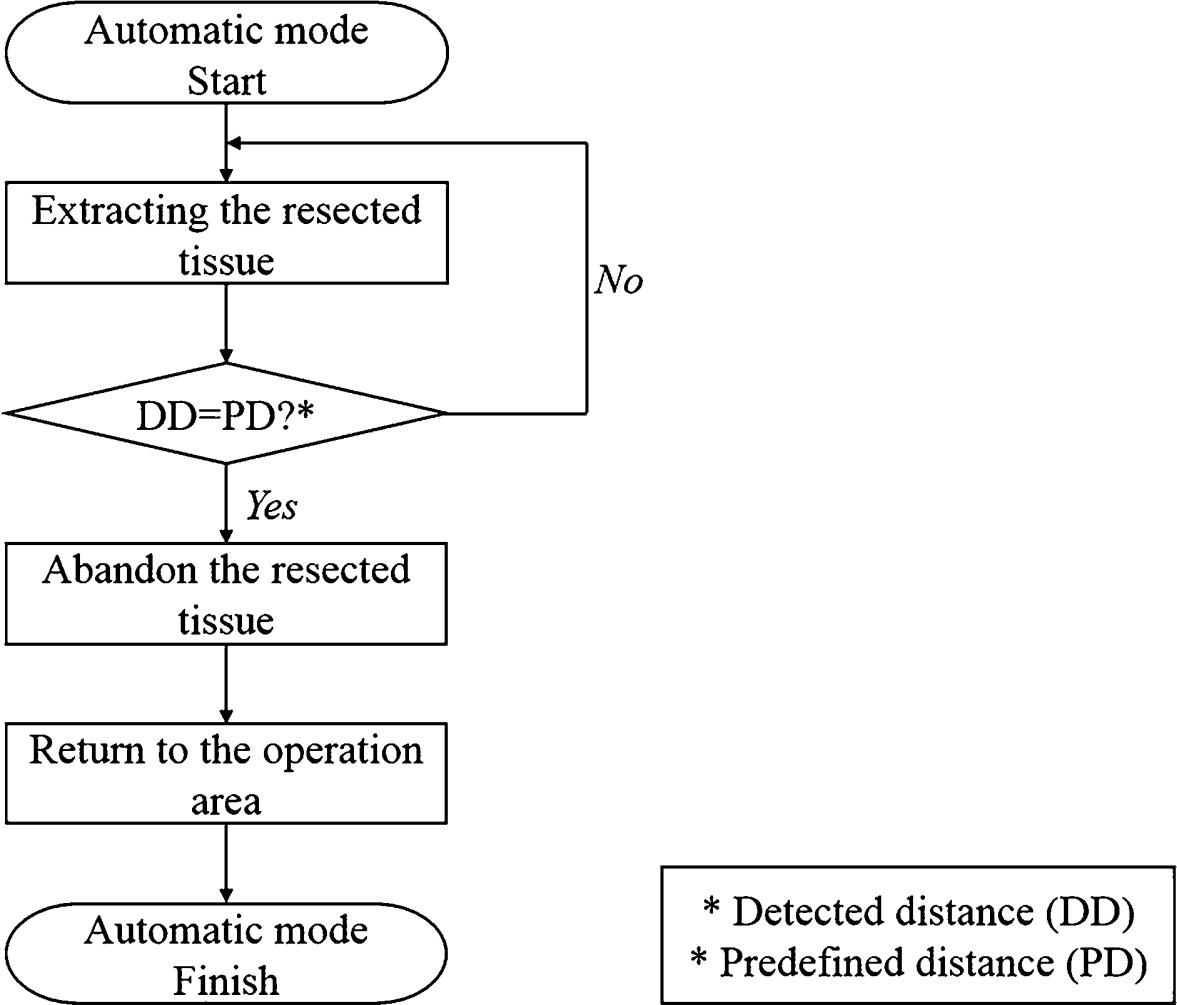
Control flow of the automatic mode needed in the in vitro test

Thus, by sensing the magnetic field generated by the surgical instrument’s built-in magnet based on the microcontroller board and the magnetic sensor, it was able to notify the system of the distance between the end of the end-effector and the simulated trocar. Then, the assistant surgical robot system was commanded to translationally move 6 cm from the moment that the detected distance equal to zero which would result in the end of the end-effector is on the simulated trocar. In such a case, the surgical instrument would abandon the resected object and return to the operation area. Consequently, the third step of the in vitro test could be operated automatically. The control flow of the automatic mode is outlined in Fig. 12.

Each volunteer repeated the in vitro test three times. All the volunteers were able to successfully remove the resected object using the assistant robot arm. Further, no collision occurred between the operation arm and the assistant arm during any of the tests.

## Discussion

Two NMIs were respectively attached to each of the MTMs of the dVRK system to enable simultaneous manipulation of the assistant surgical robot system. The accuracy of the NMIs was evaluated via an experiment that was repeated 50 times with no error occurring. The results of the latency and power consumption experiments showed that the motions of the proposed assistant surgical robot system, except for the gripping motion, are able to act on the decision of the surgeon in 132 ms via the NMI, which can be regarded as a real-time system [37], and the power capacity can cover several types of surgeries. Further, even if the power source might not be durable for the whole time of long surgeries, the NMI is still effective because its power source can be easily replaced. These experimental results demonstrate that the NMI can be used to reflect the surgeon’s decision wirelessly and to manipulate the assistant surgical robot system without errors.

The assistant surgical robot arm was developed by integrating the 6-DOFs external robot arm and the surgical instrument. The results of repeated gripping force of the surgical instrument indicate that the gripping force is comparable to that of conventional systems [2, 38, 39]. In addition, because the relationship between the micromotor’s revolution and the generated gripping force show good linearity with 1.00 of the coefficient of determination, the gripping force of the surgical instrument could be sensitively controlled by adjusting the micromotor’s position. The reaction time of the surgical instrument’s gripping motion was determined to be 400 ms. Thus, the total time delay from the surgeon giving the command to the surgical instrument actually gripping the object is 532 ms, which cannot be considered as a perfect real-time control due to its relatively long time delay. However, the gripping motion is still effective since the time delay around 500 ms is acceptable for surgical performance and can be adapted by human [40–43]. Furthermore, since the main cause of the time delay of the surgical instrument is the micromotor’s speed, which was set to 75 % of the maximum speed during the experiment, the time delay would be shorter if the speed of the micromotor was increased. The results of 1000 on and off motions to check the durability of the surgical instrument show that the effect on the surgical instrument’s force value was negligible. This experiment was adopted from previous research [2] because the durability of the surgical instrument developed cannot be tested based on the number of surgeries, as done in the case of the EndoWrist. For the final important step in the evaluation of the surgical instrument, the sterility issue has to be considered. Thus, sealing of the surgical instrument developed is planned for future work. As illustrated in Fig. 9, the workspace of the assistant robot arm was calculated using the joint information of the 6-DOFs external robot arm [2]. The cone-like shape of the calculated workspace is a result of the fulcrum point motion of the surgical robot system. Because the workspace is much larger than the cholecystectomy workspace, the assistant surgical robot is expected to be able to perform many types of surgeries whose workspaces can be covered by the cholecystectomy. Furthermore, the size of the calculated workspace can be increased by adjusting the limits of the range of movement of the 6-DOFs external robot’s arm joints.

The resulting mean time and SD of the simple peg tasks were shorter than those of other similar systems using the same FLS kit and following the same FLS peg transfer task curriculum to validate their systems, which demonstrated that a good performance and effectiveness can be provided by the proposed assistant surgical robot system [2, 34–36]. Furthermore, as shown in Table 2, the mean of each peg task’s execution time was gradually decreased. This can be interpreted that the volunteers quickly adapted to the system and therefore showed a better result trial by trial. However, the mean and SD of each peg task’s execution time were slightly longer when compared with the results using dVRK [2], which used only one MTM with one PSM and followed the same FLS peg transfer task curriculum using the same FLS peg transfer kit. The major cause of this result is the relatively slow speed of the external robot arm. Therefore, the results can be improved by developing a more stable control algorithm for the proposed system to enable higher speed.

The in vitro test of semi-automatic resected object removal indicated that the recruited volunteers were able to manipulate both of operation robot arms and assistant robot arm. Moreover, no collisions occurred during the tests. This means that using the proposed assistant surgical robot system, the surgeon can simultaneously perform the role of assistant to prevent collision between the operation robot arm and the assistant instrument.

**Fig. 13:**
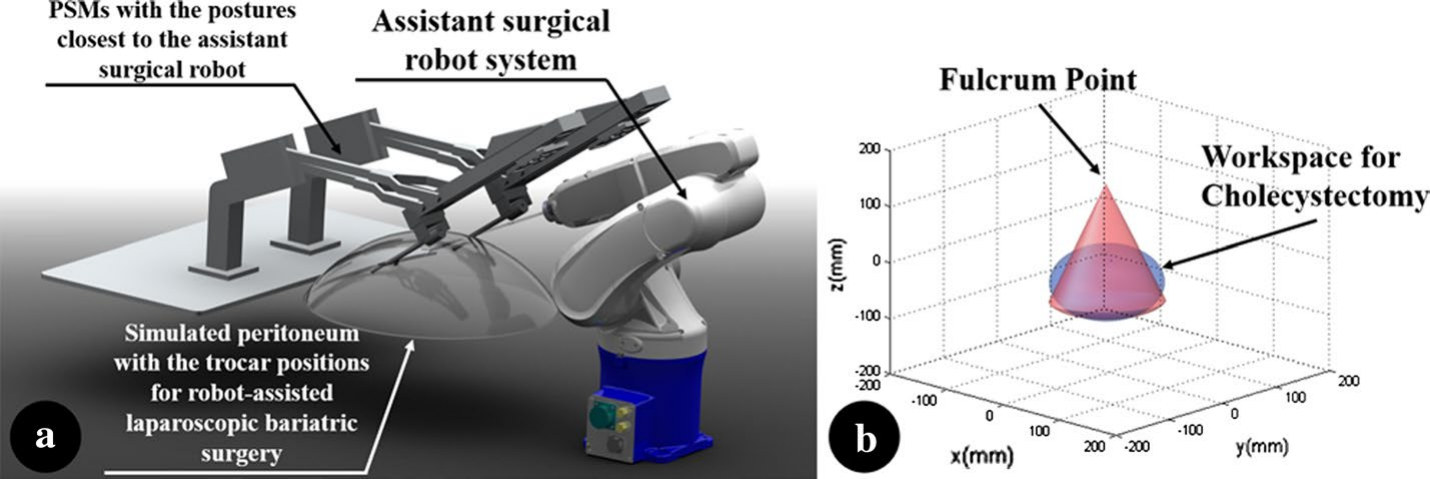
Workspace analysis of the proposed assistant robot arm system with regard to PSMs. **a** An extreme condition to the assistant robot arm system’s workspace. **b** Calculated workspace in the extreme condition

However, due to the involvement of the external robot arm, there is possibility for the collisions between PSMs and the external robot arm. Thus, there is need to calculate the external robot arm’s workspace with respect to two PSMs. For a more accurate calculation, trocar positions for robot-assisted laparoscopic bariatric surgery, which are also used for gastroesophageal reflux procedure, were used [44]. This is because the surgery also requires for a trocar used by the assistant. Furthermore, the postures of the two PSMs were set to be closest to the external robot arm for creating an extreme condition to the external robot arm’s workspace, as shown in Fig. 13a. The endoscope has been excluded from consideration since the trocar position of the endoscope is even behind those of the PSMs [44] and its posture would not be toward the PSMs considering the operation area. The workspace has been calculated based on the measurement data of the PSMs and the external robot arm, such as actual dimension and maximum angle of motion. Under the assumption that the postures of the PSMs closest to the external robot arm using the maximum angle of motion data, the external robot arm performed virtual remote center of motion while detecting the interference between the external robot arm and the PSMs. The workspace was then obtained by calculating the area without any interference which infers a collision-free area. As a result, the workspace for this condition was calculated to be 386.4 cm^3^, which could cover 70 % of the workspace for cholecystectomy, as shown in Fig. 13b. Therefore, the external robot arm’s workspace with regard to two PSMs can be also deemed to be sufficient considering the condition for above workspace was set to be extreme and it does not require for a big workspace to remove a resected object. This also implies less possibility for collision since no complex control would be commanded to an assistant surgical robot system.

Using the proposed surgical robot system, with its SOBW-type surgical instrument, NMI based on HOTAS, and the dVRK system, surgeons will be able to execute the functions of an assistant and thereby avoid collisions without having to stop surgical operations.

## Conclusions

Robot-assisted laparoscopic surgery is a very desirable surgical operation because it provides several benefits compared with open surgery and conventional MIS. However, a major issue with robotic surgery has been the collision between the operation robot arm and the assistant instrument. Consequently, this research proposed an assistant robot system that can be simultaneously manipulated by the surgeon via a wireless controller. The assistant robot arm comprises a surgical instrument with a diameter of 6 mm and 6-DOFs external robot arm. The surgical instrument uses a micromotor to generate gripping motion and the external robot arm can perform translational, fulcrum point, and rolling motions with the surgical instrument. The surgical instrument, which is based on SOBW, was validated via a gripping force experiment, a reaction time test, and a durability test. The workspace of the assistant robot system has clinical applicability. A wireless communication interface designed based on the HOTAS concept, called NMI, facilitates simultaneous manipulation of the assistant robot arm and the operation robot arm. In this study, a tiny piece of hardware was developed which is attached to the MTM of the dVRK system, which was used as the operation robot. The results of accuracy tests, data transfer time experiments, and a power consumption test have confirmed that the proposed NMI is feasible & effective. The results of a simple peg task and an in vitro test using the dVRK system have also indicated that the proposed system can be utilized in various types of laparoscopic robotic surgeries. However, the sterility issue needs to be resolved for the clinical application and this issue will be handled as future work.

## Abbreviations

MIS: minimally invasive surgery
DOFs: degrees of freedom
SOBW: surgical-operation-by-wire
FBW: fly-by-wire
dVRK: da Vinci research kit
MTM: master tool manipulator
PSM: patient side manipulator
NMI: novel master interface
HOTAS: hands-on-throttle-and-stick
RCM: remote center of motion
SD: standard deviation
FLS: fundamentals of laparoscopic surgery
